# Energy Expenditure and Changes in Body Composition During Submarine Deployment—An Observational Study “DasBoost 2-2017”

**DOI:** 10.3390/nu12010226

**Published:** 2020-01-15

**Authors:** Gerard Rietjens, Jasper Most, Peter J. Joris, Pieter Helmhout, Guy Plasqui

**Affiliations:** 1 Training Medicine and Training Physiology, Army Command/Directory of Personnel, Royal Netherlands Army, Ministry of Defence, Herculeslaan 1, 3584 AB Utrecht, The Netherlands; gerardrietjens@gmail.com (G.R.); PH.Helmhout.01@mindef.nl (P.H.); 2 Department of Human Physiology and Sports Medicine, Vrije Universiteit Brussel, Pleinlaan 2, U-Residence, Verd. 1, 1050 Etterbeek, Brussels, Belgium; 3 Department of Nutrition and Movement Sciences, NUTRIM School of Nutrition and Translational Research in Metabolism, Maastricht University Medical Centre, Universiteitssingel 50, 6229 ER Maastricht, The Netherlands; p.joris@maastrichtuniversity.nl (P.J.J.); g.plasqui@maastrichtuniversity.nl (G.P.)

**Keywords:** submarine, energy expenditure, doubly labelled water, body composition, adiposity, physical activity

## Abstract

The present study was designed to objectively assess the effects of 3-months submarine deployment on behavioural and metabolic determinants of metabolic health. In 13 healthy, non-obese volunteers, we using stable isotope dilution, and plasma and urinary biochemistry to characterize metabolic health before and after a 3-month submarine deployment. Volunteers worked in 6-h shifts. After deployment, we observed reduced fat-free mass (mean ± SD, −4.1 ± 3.3 kg, *p* = 0.003) and increased adiposity (21.9 ± 3.2% fat mass to 24.4 ± 4.7%, *p =* 0.01). Changes in fat-free mass were positively associated with physical activity (+0.8 kg per 0.1 increase in PAL, *p =* 0.03). The average physical activity level was 1.64 ± 0.26 and total energy expenditure during deployment was 2937 ± 498 kcal/d, while energy intake was 3158 ± 786 kcal/d. Fasting glucose (*p =* 0.03), and triglycerides (*p =* 0.01) declined, whereas fasting free fatty acids increased (*p =* 0.04). Plasma vitamin D and B12 concentrations decreased (−14%, *p =* 0.04, and −44%, *p =* 0.001, respectively), and plasma calcium, and magnesium increased (+51%, *p =* 0.01, and +5%, *p =* 0.02). Haemoglobin was unchanged, but haematocrit decreased (−2.2 ± 2.1%, *p =* 0.005). In conclusion, submarine deployment impairs fat-free mass maintenance and promotes adiposity. High physical activity may prevent the decline in fat-free mass. Our study confirms the need to counteract Vitamin D and B12 deficiencies, and suggests impairments in erythrocyte metabolism.

## 1. Introduction

Physical inactivity and adiposity impair metabolic, musculoskeletal and mental health. In the military, these factors affect performance and military readiness. Indeed, reduced mass and strength of the musculoskeletal apparatus increases the risk for injuries and fatalities [1,2], and impaired metabolic health may negatively affect endurance, resiliency, and recovery [3,4].

During military deployments, habitual activity and dietary patterns are affected by living environment, access to food, assignments and schedules, or climate. Specifically, deployment settings onto submarines reduce opportunity for activity, void access to fresh foods and sunlight, and require shift-work of 6 h on-duty and 6 h off-duty. As a result, submarine deployment has been shown to impair metabolic health, including promotion of obesity, metabolic syndrome and reduced bone health [5,6,7,8,9,10,11].

We hypothesize that these effects may be mediated by a positive energy balance, e.g., low physical activity and overeating, and may therefore be preventable. However, to date, no study has assessed energy balance, obtained objectively using doubly labelled water, and adverse effects of submarine deployment on cardiometabolic health.

We therefore performed a comprehensive assessment of anthropometric, metabolic and behavioural variables that serves to understand the impact of submarine deployment on metabolic health. Ultimately, this study may assist to inform strategies to maintain metabolic health and fitness during deployment.

## 2. Materials and Methods

### 2.1. Design

In this observational study, we assessed the effect of a 3-month deployment onto a submarine on anthropometric, cardiovascular, metabolic and behavioural outcomes in 13 male submarine soldiers. This study was approved by the Staff Joint Health Care Division of the Dutch Ministry of Defence (“Dasboost2-2017”). The investigations were carried out following the rules of the Declaration of Helsinki of 1975, revised in 2013, and all participants signed informed consent prior to initiation of any study procedures.

Before and after employment (week 0 and week 12), we measured body composition by deuterium dilution, cardiovascular risk factors, including blood pressure and fasting plasma lipids, and measures of metabolic and skeletal health in blood and urine. During deployment (week 4 and 5 of deployment), total daily energy expenditure was measured over a period of two weeks using doubly-labelled water. Daily energy intake and energy balance were assessed using the energy intake-balance methods [12,13], using energy expenditure as measured during week 4 and 5 of deployment, and changes in body composition assessed before and after 3 months of deployment.

### 2.2. Deployment

The deployment period onto the submarine covered 3 months. During deployment, participants worked in 6 hours-shifts. Participants consumed three meals a day, and had free access to snacks outside of regular meals. Participants were not given any instruction or advice on nutrition or activity for this study and were free to use dietary supplements. On average, participants slept 5:46 ± 1:28 h per day (personal communication). Within the confined environment in the submarine, we measured an average oxygen concentration of 20.24% (19.64 to 20.62%) and carbondioxide concentration of 0.304% (range, 0.058 to 0.647%). Gas concentrations were variable over the day but on average remained constant over the time of deployment.

### 2.3. Measurements

At recruitment, volunteers completed a comprehensive lifestyle assessment questionnaire, to assess, amongst others, questions on habitual physical activity, smoking, intake of dietary supplements, and alcohol consumption.

Body weight was measured in underwear clothing after an overnight fast. Body composition was determined by measuring total body water. After collecting two urine samples for measuring background enrichment, participants consumed 0.1 gram of 99.9% D2O dilution per kg body weight. Total body water was estimated using the plateau method 8 h after ingestion of the Deuterium dilution. Deuterium dilution space was assumed to be 4% larger than TBW. To estimate fat mass, we used FM [kg] = BW [kg] − TBW [L]/0.732, in which FM is fat mass, BW is body weight, and TBW is total body water, and 0.732 is the constant of fat-free mass hydration [14].

Blood pressure was measured in a seated upright/supine position, after participants rested for at least 5 min. Mean arterial blood pressure (MAP) was calculated as MAP = (2 × DBP + SBP)/3, in which DBP is diastolic blood pressure, and SBP is systolic blood pressure. Measures were performed twice and the mean of both measures was used for calculations.

We estimated the 10-year cardiovascular risk score as described by Anderson et al. [15], which is based on smoking status, history of diabetes, abnormal echocardiogram, gender, age (all unchanged during deployment), total cholesterol to HDL-ratio, and systolic blood pressure. The changes in CVD-risk would be unaffected by consideration of the former set of variables, because they did not change over time; before and after the intervention, the risk would be affected by the following factors as follows: smoking +2.0%, history for diabetes: +1.55+, abnormal echocardiogram: +3.99%.

Skinfold thickness was measured twice at four sites each (biceps, triceps, subscapular, and suprailiac).

Blood and urine samples were collected after an overnight fast. For blood cell analyses, whole blood was used. Plasma samples were obtained in EDTA-tubes after immediate centrifugation at 10,000 rpm and 4 °C for 10 min. Serum samples were obtained by centrifugation (10,000 rpm, 21 °C, 15 min), after blood was allowed to clot for 90 min at room temperature. Aliquots were snap-frozen in liquid nitrogen before storage at −80 °C until further analysis. Urine samples were collected into containers and stored at −20 °C, and aliquots were snap-frozen in liquid nitrogen and stored at −80 °C until further analysis.

Total daily energy expenditure was measured using the doubly-labelled water technique [16]. Three weeks after deployment, fasting urine samples were collected to assess baseline isotope enrichments. According to the Maastricht protocol, participants received on average a dose of 114 ± 11 g 6% D_2_O and 10% H_2_^18^O. Total body water was estimated as described. Carbondioxide production was calculated from the difference in elimination rates of deuterium and 18O using equation A6 from Schoeller et al. [17]. Energy expenditure was calculated per Weir et al. [18], by assuming a respiratory quotient (RQ) of 0.85, reflecting an average Western diet (15%, 40%, and 45% energy from protein, fat and carbohydrates) [19]. Since the measurements were performed in a confined environment, we assessed whether the isotopes were enriched in the atmosphere and would bias the dilution estimates. To this end, parallel to the measurement of isotope enrichments in study participants we measured the background enrichment of both isotopes 15 subjects, who were on the same submarine during deployment, yet not part of this study. We observed no increase in background enrichment of the isotopes. The physical activity level (PAL) was defined as the ratio between TDEE and BMR. BMR was derived from the Harris-Benedict-equation [20]. Daily energy intake was calculated as the sum of TDEE and the daily changes in energy storage in fat mass (13.1 kcal/g) and fat-free mass (2.2 kcal/g) [12,13], derived from body composition measurements before and after deployment (10 weeks).

### 2.4. Statistics

Data are expressed as mean ± SD. The present study is exploratory and sample size is limited to the capacity of the submarine as well as the engagement of the crew. Therefore, no a priori power calculation was performed. Instead, we performed retrospective power analysis based on the changes in the primary outcome, which are the changes in fat mass and changes in fat-free mass. Changes over time were tested by paired t-tests. Normality of baseline values was confirmed by Shapiro-Wilk-tests. To assess determinants of changes in body composition, we performed stepwise linear regression with the changes in fat mass and fat-free mass as dependent variables. Independent variables were baseline body size, physical activity level (PAL) and energy intake. Next, we assessed whether the residuals of the produced model were correlated with observed, significant changes in metabolic biomarkers. Statistical significance was considered if *p* < 0.05. Statistical analysis was performed using SPSS software, Version 25 for Mac, Version 10 (IBM Corp, Armonk, NY, USA). 

## 3. Results

### 3.1. Subject Characteristics

A total of 55 men were deployed on this mission, of which 13 volunteers enrolled in this study (27.8 ± 5.8 years, 24.8 ± 3.7 kg/m^2^). Complete body composition data was available for ten (28.0 ± 6.7 years, 25.6 ± 3.7 kg/m^2^); body composition measurements were unsuccessful in two participants at baseline (no isotope enrichment, and not dosed) and one provided unreliable results after deployment (i.e., estimated body fat of 2%).

### 3.2. Body Composition and Energy Balance

**Figure 1:**
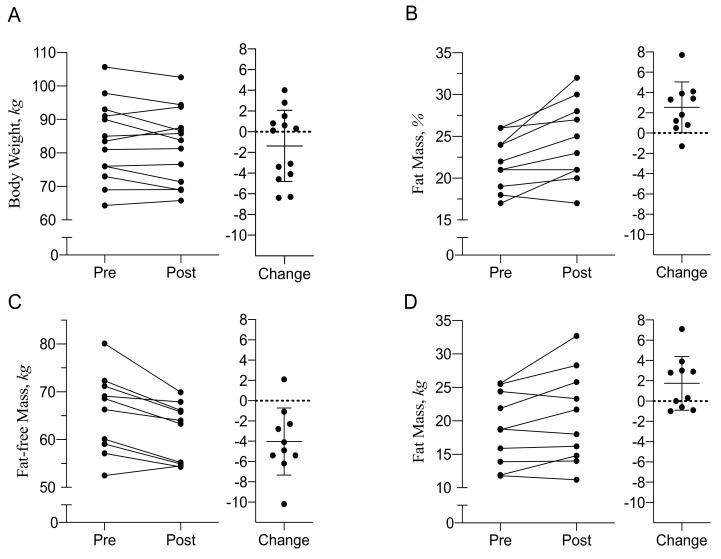
Changes in Body Weight and Body Composition after 10-Week Deployment on a Submarine. Individual changes in body weight (**A**, *n* = 13) and body composition (**B**–**D**, *n* = 10) before and after 10-week deployment on a submarine are presented. The changes are also presented as mean ± SD.

During deployment, participants maintained their body weight (*n* = 13, −1.4 ± 3.4 kg, *p* = 0.18, range +4.0 to −6.4 kg, Figure 1A). Measurements of body composition (*n* = 10) showed a significant increase in adiposity (21.9 ± 3.2% fat mass to 24.4 ± 4.7%, *p* = 0.01, Figure 1B). This increase was caused by a reduction in fat-free mass (−4.1 ± 3.3 kg, *p* = 0.003, Figure 1C), which occurred in all participants, except for one. Fat mass increased, but not statistically significant (+1.8 ± 2.3 kg, *p* = 0.06, Figure 1D). With an appropriate control group (assuming changes equal to 0), a randomized study would require a sample size of 66 subjects to detect the observed change in fat mass (1.8 ± 2.6 kg) with adequate power (>80%) and a significance level of *p* < α, with α < 0.05), and a sample size of 22 subjects for the change in fat-free mass (−4.1 ± 3.3 kg).

Skinfold thickness increased by 14 ± 13 mm during deployment (35%, *p* = 0.002, estimated fat mass: +3.3 ± 2.9%, *p* = 0.001). Most pronounced changes were observed at the iliac crest (+8.9 ± 7.8 mm, i.e., +55%, *p* = 0.001), in addition, increases at the triceps were significant (+1.0 ± 0.4 mm, i.e., 15%, *p* = 0.04).

During deployment, total daily energy expenditure of all measured participants (*n* = 13) was 3315 ± 560 kcal/d and physical activity level was 1.64 ± 0.26 (range, 1.29 to 2.11). For participants with body composition-data available (*n* = 10), we assessed energy balance using the intake-balance method. Total daily energy expenditure in this group was 2937 ± 498 kcal/d, physical activity level 1.54 ± 0.21 (1.29 to 1.94), daily energy intake was 3158 ± 786 kcal/d, and as a result, energy balance tended to be positive (+221 ± 506 kcal/d, *p* = 0.10).

### 3.3. Determinants of Changes in Fat Mass and Fat-Free Mass

**Figure 2:**
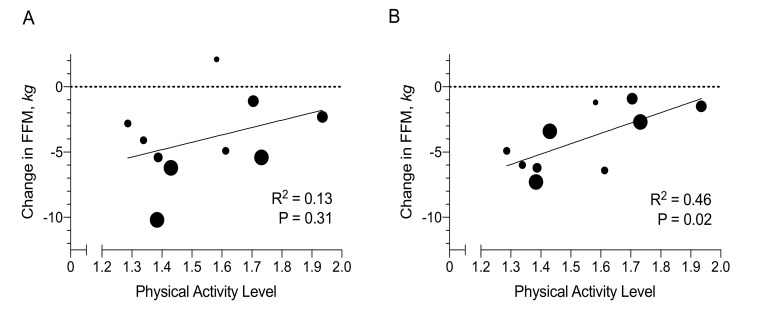
Physical Activity and Changes in Fat-free Mass. The association between the physical activity level and change in fat-free mass during a 3-month deployment onto military submarine is presented before (**A**) and after (**B**) adjustment for body mass index (*n* = 10). Larger size of each data point indicates higher baseline BMI (range from 20.0 to 30.2 kg/m^2^). The association between physical activity level and change in fat-free mass was assessed by linear regression.

Changes in fat-free mass were negatively associated with pre-deployment body size (BMI, *n* = 10, *r* = −0.68, *p* = 0.03), body weight (*r* = −0.74, *p* = 0.02), fat mass (*r* = −0.65, *p* = 0.04) and fat-free mass (*r* = −0.77, *p* = 0.01). Changes in fat-free mass only associated positively with physical activity level after adjustment for BMI (0.8 ± 0.3 kg of fat-free mass was lost less per 0.1 increase in PAL, *p* = 0.03, Figure 2A,B). Therefore, to maintain fat-free mass would require a PAL that is dependent on BMI: PAL = (0.7 × BMI [kg/m^2^] − 1.1)/8.375, *R*^2^ = 0.73, *p* = 0.01.

The change in fat mass was not associated with pre-deployment BMI (*r* = 0.48, *p* = 0.16). To estimate energy requirements for body weight maintenance, i.e., to avoid fat accumulation, we performed a linear regression with total daily energy expenditure as dependent variable, and body composition and physical activity as independent variables:

TDEE [kcal/d] = 20.1 × BW [kg] + 1939 × PAL − 1737, *R*^2^ = 0.99, *p* < 0.001 (1)

TDEE [kcal/d] = 31.5 × FFM [kg] + 3.4 × FM [kg] + 1941 × PAL − 2184, *R*^2^ = 0.99, *p* < 0.001 (2)

### 3.4. Cardiometabolic Risk Markers

Changes in cardiometabolic risk markers are summarized in Table 1. Plasma glucose decreased by 0.3 ± 0.4 mmol/L (*p* = 0.03) and serum insulin concentrations tended to decrease (−1.5 ± 2.7 mU/mL, *p* = 0.07) during deployment. Estimates for both insulin resistance (HOMA-IR) and insulin secretion (HOMA-b) improved. Fasting free fatty acids were increased after deployment (+0.25 mmol/L, *p* = 0.04), while triglyceride concentrations decreased (−0.3 ± 0.4 mmol/L, *p* = 0.01). Besides a trend decrease in SBP, other cardiometabolic risk markers (i.e., blood pressure, total cholesterol, HDL- or LDL concentrations) did not change. The estimated 10-year-cardiovascular risk score was therefore unchanged after 10 weeks (−0.04 ± 0.3%, *p* = 0.66).

Plasma and urinary micronutrient and electrolyte concentrations before and after deployment are summarized in Table 2. After exclusion of a subject that started using Vitamin D supplements during deployment (plasma concentration increased by 46%), Vitamin D concentrations significantly decreased (−11 ± 5%, *p* = 0.04). Also, we observed significant increases in plasma Calcium and Magnesium concentrations, while their concentrations in urine decreased. Plasma concentrations of parathyroid hormone, alkaline phosphatase, or phosphorus concentrations did not change. Lastly, we observed significant changes in hematocrite, and blood concentrations of ferritine, folic acid and vitamin B12. Changes in plasma and urinary micronutrient and electrolyte concentrations were not associated with changes in body composition.

## 4. Discussion

To understand the impact of a prolonged deployment, in a confined environment, on a 6-h day/night schedule, and without sunlight, on energy metabolism and metabolic health, we have provided a comprehensive assessment of behavioural and metabolic factors that may compromise military readiness. As hypothesized, we observed significant changes in body composition during a 3-month military deployment onto a submarine. Most strikingly, participants lost 4 kg of fat-free mass. Using gold-standard methodologies of biomedical research, we demonstrated that this reduction in fat-free mass causes a reduction in body weight despite subjects being in positive energy balance.

The average physical activity of study participants was lower than the average physical activity of the general population (Netherlands, PAL of 1.7) [21]. Therefore, low physical activity may have contributed to the loss of fat-free mass [22]. However, we estimated that, after adjustment for baseline BMI, a preservation of fat-free mass during submarine deployment requires a physical activity level of ~2.0, which is significantly higher than physical activity sufficient to maintain fat-free mass in free-living conditions [23,24]. This data therefore suggests, that submariners are at increased risk for muscle mass loss. The causes of the accelerated reduction in fat-free mass remain speculative, but may relate to the irregular daily rhythm induced by shift-work [25,26], voided sunlight/vitamin D deficiency [27,28], chronic stress [10], and altered dietary pattern, e.g., low protein [29], low eating frequency [30,31]. Notably, in other studies on submariners, a reduction in fat-free mass was not observed [7,32], and vitamin D supplementation did not increase fat-free mass during 3-month submarine deployment [6]. Importantly, in this latter study, vitamin D supplementation did not affect plasma Vitamin D concentrations differently between control and supplementation-groups.

Maintaining or reducing fat mass for participants during submarine deployment in our cohort (*n* = 10, 83 kg, 25 kg/m^2^, PAL: 1.54) requires to limit energy intake to their energy expenditure, which was 2937 kcal/d or 35 kcal per kg body weight per day. This estimate is comparable to findings from a study in 89 volunteers during a 12-week deployment of the British Royal Navy, which objectively measured energy requirements using doubly labelled water (~38 kcal/kg/d [32]), but larger than from another study, in which energy requirements were estimated in a group of 53 US volunteers during 3-months submarine deployment based on self-report with a reporting bias of ~20% (27–31 kcal/kg/d) [7]. In comparison to the present cohort, volunteers of the British study were more active (PAL of 1.8–1.9). Overall, the gain in fat mass in our study was not statistically significant, yet half of the participants gained more than 3 kg of fat mass during deployment. Over a 3-month period, such an increase translates into a positive energy balance of ~300 kcal/d. Physical activity levels were not associated with changes in fat mass, and therefore, an increase in energy intake likely caused the increase in adiposity. Skinfold measurements indicate that fat mass accumulation occurred primarily in the subcutaneous depots.

The observed reduction in body weight during positive energy balance is counterintuitive and highlights the importance of assessing body composition. Energy balance is the sum of changes in energy stores in the body and positive energy balance implies that overall, more energy is stored than released. The distinction into different body components is important, because (1) body components have different energy densities and (2) energy balance can differ per body component. In the present study, fat mass increased by 1.8 kg, which reflects energy storage of 23,000 kcal (1.8 kg × 13,100 kcal/kg fat mass). During the same period, fat-free mass decreased by 4 kg, which reflects energy release of ~9000 kcal/d (−4 kg × 2200 kcal/kg fat-free mass). Thus, while body mass decreased by 2.2 kg as the sum of changes in fat mass (+1.8 kg) and fat-free mass (−4 kg), energy balance was positive (+14,000 kcal, or 220 kcal/d), as the sum of changes in fat mass (+23,000 kcal, or 360 kcal/d) and fat-free mass (−9000 kcal or 140 kcal/d).

Plasma glucose and insulin concentrations decreased during deployment, resulting in reduced whole-body insulin resistance, assessed by HOMA-IR. Reducing insulin resistance may ameliorate the decline in muscle mass [33], and reduce the risk for type 2 diabetes development in the long term [34]. Furthermore, plasma triglyceride concentrations were lower after deployment, reducing the risk for ectopic fat accumulation and ultimately type 2 diabetes and cardiovascular diseases [35]. Moreover, fasting free fatty acids increased, which suggest less inhibition of peripheral lipolysis, in line with lower insulin concentrations. Blood pressure and serum lipoprotein concentrations were unchanged during deployment. These observations are similar to those observed in US submariners after 3 months-deployment [7], which were reported to have improved serum lipids. However, in the US-study, a reduction in body weight and fat mass was observed. Importantly, the US-cohort was significantly more obese and, therefore, more likely to lose fat mass during deployment. Indeed, larger declines in fat mass were observed in obese participants as compared to non-obese participants.

The causes for the improvements in the cardiometabolic profiles found in our study are unclear. Changes in metabolic biomarkers were not associated with changes in body composition, which may relate to the small power of this study. Unfortunately, dietary factors, including diet quality and eating behavior, and physical activity were not assessed prior to deployment. It may be speculated that dietary intake improved during deployment compared to before, because all meals were provided, and access to snacks or fast-food was limited/voided. Alcohol consumption appeared to be unrelated to these changes in glucose homeostasis. In a pre-deployment survey among submarine personnel, 98% reported alcohol consumption on a weekly base, with, on average, 7 consumptions per week, and was comparable to alcohol consumption during deployment (10 grams per day).

As expected [36], we observed a significant reduction in plasma Vitamin D concentrations. Deficient vitamin D concentrations (< 80) were observed in 8 (67%) participants after deployment, as compared to 2 (17%) before deployment. This reduction was not associated with changes in parathyroid hormone, alkaline phosphatase or plasma phosphorus concentrations.

The reduction in Vitamin D concentration is likely caused by the voided sunlight during deployment. The effects of reduced Vitamin D concentrations remain unclear, but may include calcium and bone metabolism. Vitamin D stimulates intestinal calcium absorption and bone resorption and thus, low vitamin D concentrations may impair bone remodeling. In the present study, we observed lower urinary concentrations of calcium and potassium indicating increased re-absorption of these minerals in the kidneys, which may compensate for low intestinal absorption. In a French cohort [36], deployment onto a submarine induced Vitamin D-deficiency and impaired bone remodeling. In contrast, no effects of submarine deployment, or rather some improvements, were observed on bone mass or strength in another study [6], designed to determine the effect of 400 IU/d-Vitamin D supplementation in 53 US Submariners. In this US study [6], and in a study of French Submariners [37], supplementation with Vitamin D (1000 or 2000 IU/day) has been insufficient to prevent the decline in Vitamin D concentrations and has not improved bone metabolism.

Lastly, we observed significant reductions in Vitamin B12 concentrations, suggesting Vitamin B12 deficiency. Vitamin B12 deficiency induces macrocytic anemia [38], which we did not assess in this study. Interestingly, hemoglobin, a measure of anemia, was not affected by submarine deployment, but high intakes of folic acid from fortified food and dietary supplements, as consumed during submarine deployments and evidenced by increased folic acid concentrations, might mask the macrocytic anemia of vitamin B12 deficiency [39,40]. Anemia develops gradually, so a person may not notice any symptoms until it is severe. Symptoms may include weakness, exhaustion, low appetite, confusion, paleness, redness or swelling of the tongue (glossitis), diarrhea, depression, or infertility. Such adverse events have not been documented in our study, and to our knowledge, this metabolic risks for soldiers in military (submarine) deployments has never been reported.

This study has limitations. First, the specific nature of this study limits the available sample size. Low numbers of participants and certain levels of missing data are not unusual in studies in which soldiers in combat (training) operations are monitored during extensive periods of time. Since our study took place during an operational mission, no researchers were allowed onboard to guide participants through their measurement protocol. Evidently, first priority of the crew was the execution of their operational task, which may have affected the measurements to a certain extent. Second, while the effects on body composition and metabolic biomarkers are convincing, we did not recruit a control group. Third, the study did not include measurements of eating behavior and physical behavior prior to deployment. Changes in these behavioral factors onboard may have contributed to the observed changes in body composition and metabolic biomarkers. Last, resting metabolic rate was estimated using the Harris-Benedict-equation, but not measured. The estimates were confirmed using equations considering body composition, i.e., Cunningham-equation: RMR = 500 + 22 × fat-free mass [kg].

## 5. Conclusions

In this observational study, we show that submarine deployment impairs fat-free mass maintenance and may promote adiposity. Increasing physical activity may prevent the decline in fat-free mass, but requires higher levels than observed in this study. Unexpectedly, the cardiometabolic profile of crew members improved during deployment, which may relate to diet quality, although this interpretation is speculative. Our study confirms the need to counteract Vitamin D deficiency, and impairments in erythrocyte metabolism. Lastly, this study suggests the need for post-deployment interventions designed to foster recovery and thereby avoid adverse long-term effects including adiposity, and improve military readiness for subsequent missions.

## Figures and Tables

**Table 1:** Cardiometabolic risk markers.

	Pre	Post	Change		*p*
Glucose Homeostasis					
Glucose, mmol/L	5.4 ± 0.4	5.2 ± 0.5	−0.3 ± 0.4	−5%	0.03
Insulin, mU/L	5.0 ± 4.8	3.5 ± 2.7	−1.5 ± 2.7	−30%	0.07
HOMA-IR	1.3 ± 1.3	0.8 ± 0.7	−0.4 ± 0.8	−34%	0.06
HOMA-ß	14.2 ± 16.0	9.7 ± 10.0	−4.5 ± 8.7	−32%	0.09
Cardiovascular Biomarkers					
Triglycerides, mmol/L	1.2 ± 0.5	0.9 ± 0.3	−0.3 ± 0.4	−27%	0.01
Free fatty acids, mmol/L	0.3 ± 0.2	0.6 ± 0.3	0.3 ± 0.4	76%	0.04
Cholesterol, total, mmol/L	4.5 ± 0.6	4.5 ± 0.6	−0.05 ± 0.6	−1%	0.79
Cholesterol, HDL, mmol/L	1.4 ± 0.3	1.3 ± 0.3	−0.1 ± 0.2	−7%	0.10
Cholesterol, LDL, mmol/L	2.6 ± 0.7	2.8 ± 0.6	0.2 ± 0.5	8%	0.21
Systolic blood pressure, mmHg	123.0 ± 8.7	120.0 ± 9.0	−3.0 ± 4.5	−2%	0.08
Diastolic blood pressure, mmHg	75.2 ± 6.5	77.8 ± 5.6	2.6 ± 8.2	3%	0.38
Mean arterial blood pressure, mmHg	91.2 ± 4.4	92.0 ± 5.7	0.8 ± 6.1	1%	0.71
10-year CVD risk, %	0.9 ± 1.3	0.9 ± 1.1	−0.04 ± 0.3	−5%	0.66

Data are reported as Mean ± SD and as % for 13 participants (9 for blood pressure). Changes over time (pre to post) were assessed by paired Student’s *t*-Test, and were considered significant if *p* < 0.05. CVD, cardiovascular diseases; HOMA-IR, homeostatic model assessment of insulin resistance; HOMA-ß, homeostatic model assessment of insulin secretion.

**Table 2:** Plasma and Urinary Biochemistry.

	N	Pre	Post	Change		*p*
P Vitamin D3, nmol/L	12	200 ± 41	173 ± 35	−25 ± 39	−14%	0.04
P Calcium, mmol/L	11	2.4 ± 0.1	3.6 ± 1.5	1.2 ± 1.5	51%	0.02
U Calcium, mmol/L	11	4.6 ± 2.2	2.5 ± 0.1	−2.1 ± 2.2	−45%	0.01
P Magnesium, mmol/L	13	0.83 ± 0.06	0.86 ± 0.06	0.04 ± 0.05	5%	0.02
U Magnesium, mmol/L	11	5.2 ± 1.7	4.2 ± 1.4	−1.1 ± 2.1	−20%	0.12
P Parathyroid Hormone, mmol/L	13	2.8 ± 0.8	2.7 ± 1.5	−0.1 ± 1.1	−3%	0.80
P Alkaline Phosphatase, IU/L	13	76 ± 17	77 ± 16	1 ± 9	1%	0.80
P Phosphorus, mmol/L	13	1.1 ± 0.1	1.1 ± 0.2	0.1 ± 0.2	5%	0.29
U Phosphorus, mmol/L	11	31 ± 8	37 ± 10	6 ± 12	18%	0.17
P Sodium, mmol/L	13	141 ± 1	141 ± 1	0.3 ± 1.8	0%	0.56
U Sodium, mmol/L	11	128 ± 73	141 ± 52	13 ± 89	10%	0.63
P Potassium, mmol/L	13	4.5 ± 0.2	4.4 ± 0.2	−0.1 ± 0.3	−2%	0.33
U Potassium, mmol/L	11	57 ± 22	46 ± 13	−11 ± 16	−19%	0.05
P Chloride, mmol/L	13	105 ± 2	104 ± 1	−1 ± 2	−1%	0.02
U Chloride, mmol/L	11	143 ± 84	129 ± 56	−14 ± 92	−10%	0.63
B Haemoglobin, mmol/L	12	9.4 ± 0.8	9.4 ± 0.7	0.03 ± 0.39	0%	0.77
B Haematocrit, %	12	46.6 ± 3.3	44.4 ± 2.1	−2.2 ± 2.1	−5%	0.005
B Ferritin, ng/mL	12	122 ± 60	200 ± 127	78 ± 75	64%	0.004
B Vitamin B12, pmol/L	12	281 ± 95	158 ± 65	−123 ± 98	−44%	0.001
B Folic Acid, ng/mL	11	5.5 ± 1.5	13.1 ± 5.7	7.5 ± 5.8	136%	0.002
U Osmolality, mOls/kg	11	846 ± 225	882 ± 209	36 ± 201	4%	0.56
U Creatinine, mmol/day	11	19 ± 11	21 ± 6	2 ± 12	10%	0.60
U Urea, mmol/L	11	418 ± 122	469 ± 137	50 ± 103	12%	0.14

Data are reported as Mean ± SD. Changes over time (pre to post) were assessed by paired Student’s *t*-Test, and were considered significant if *p* < 0.05. P, Plasma; U, Urinary.
